# Insulin stimulated MCF7 breast cancer cells: Proteome dataset

**DOI:** 10.1016/j.dib.2016.09.025

**Published:** 2016-09-22

**Authors:** Hetal A. Sarvaiya, Iulia M. Lazar

**Affiliations:** Department of Biological Sciences, Virginia Tech, 1981 Kraft Drive, Blacksburg, VA 24061, USA

**Keywords:** MCF7 breast cancer cells, Proteomics, Mass spectrometry

## Abstract

The proteome data provided in this article were acquired from MCF7 breast cancer cells stimulated with insulin, and were generated by using a 2D-SCX (strong cation exchange)/RPLC (reversed phase liquid chromatography) separation protocol followed by tandem mass spectrometry (MS) detection. To facilitate data re-processing by more advanced search engines and the extraction of additional information from already existing files, both raw and processed data are provided. The sample preparation, data acquisition and processing protocols are described in detail. The raw data relate to work published in “Proteome profile of the MCF7 cancer cell line: a mass spectrometric evaluation” (Sarvaiya et al., 2006) [1] and are made available through the PRIDE (PRoteomics IDEntifications)/ProteomeXchange public repository with identifier PRIDE: PXD004051 (“2016 update of the PRIDE database and tools” (Vizcaino et al., 2016) [2]).

**Specifications Table**

**Table:** 

Subject area	*Chemistry, Biology*
More specific subject area	*Proteomics*
Type of data	*Excel files, figures.*
How data was acquired	*Data were generated by data-dependent LC-MS/MS analysis using an 1100 HPLC system (Agilent) interfaced to an LTQ ion trap mass spectrometer (Thermo Electron).*
Data format	*Raw, processed, analyzed.*
Experimental factors	*MCF7 breast cancer cells cultured in EMEM with insulin (10 µg/mL) and FBS (10%).*
Experimental features	*Cells were harvested at 70–80% confluence, lysed in RIPA buffer, digested with trypsin and analyzed by 2D-SCX/C18 reversed phase nano-LC and ESI-MS/MS.*
Data source location	*Virginia Tech, Blacksburg, VA 24061, USA.*
Data accessibility	*Data are provided in this article and MS RAW and processed files have been deposited in the ProteomeXchange Consortium via the PRIDE partner repository with the dataset identifier PRIDE*[2]:PXD004051 and DOI: 10.6019/PXD004051.

**Value of the data**

• The data provided in this manuscript describe the proteome profile of insulin-stimulated MCF7 breast cancer cells.
• The MS RAW files can be used to verify the fragmentation pattern of 1+, 2+ and 3+ non-labeled peptide ions in a linear ion trap analyzer, to experimentally confirm computational predictions, and to select precursor-fragment transitions for MRM method development.
• The MS RAW files can be re-processed with more advanced (or, a combination) of search engines, to enable the identification of additional peptides and proteins, and to confirm the expression of certain proteins under insulin stimulation conditions.
• The biological processes, functional categories and signaling pathways that were identified in these cells can be used as a reference for comparison with other cell stimulation conditions, or for validating data generated in other laboratories and support the identification of putative drug targets or biomarkers.

## 1 Data

**Fig. 1:**
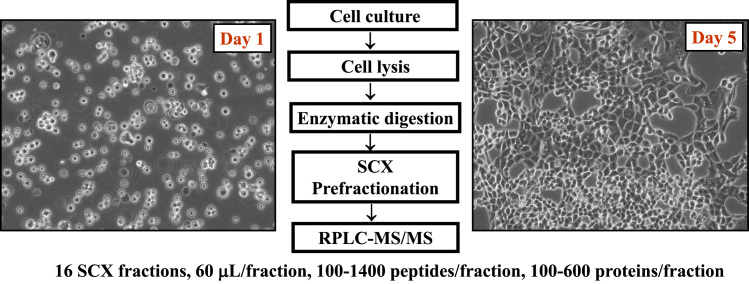
Outline of the MCF7 cell extract processing protocol.

**Fig. 2:**
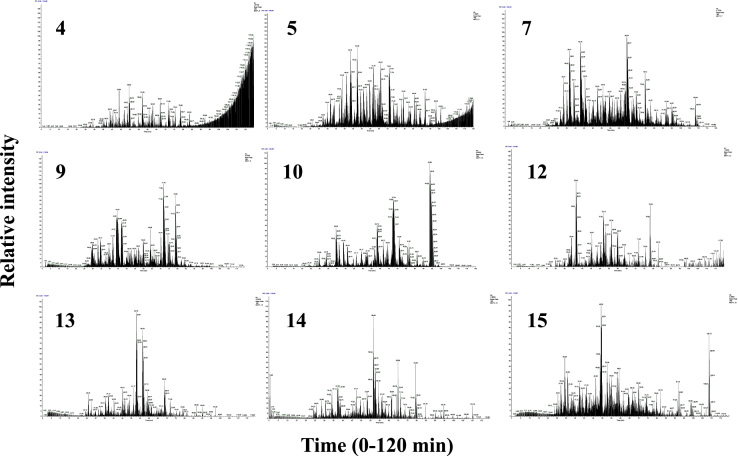
Representative base-peak chromatograms for 16 SCX peptide fractions generated from MCF7 cell extracts. The LC and MS systems were operated independently of each other, with a window of ~1–5 min for turning on the MS data acquisition after the LC separation start. Most peptides eluted in fractions 4–15. Fractions 1 (SCX wash), 2, 3 and 16 contained salts, traces of detergents and contaminants from sample preparation, and only few peptides.

**Fig. 3:**
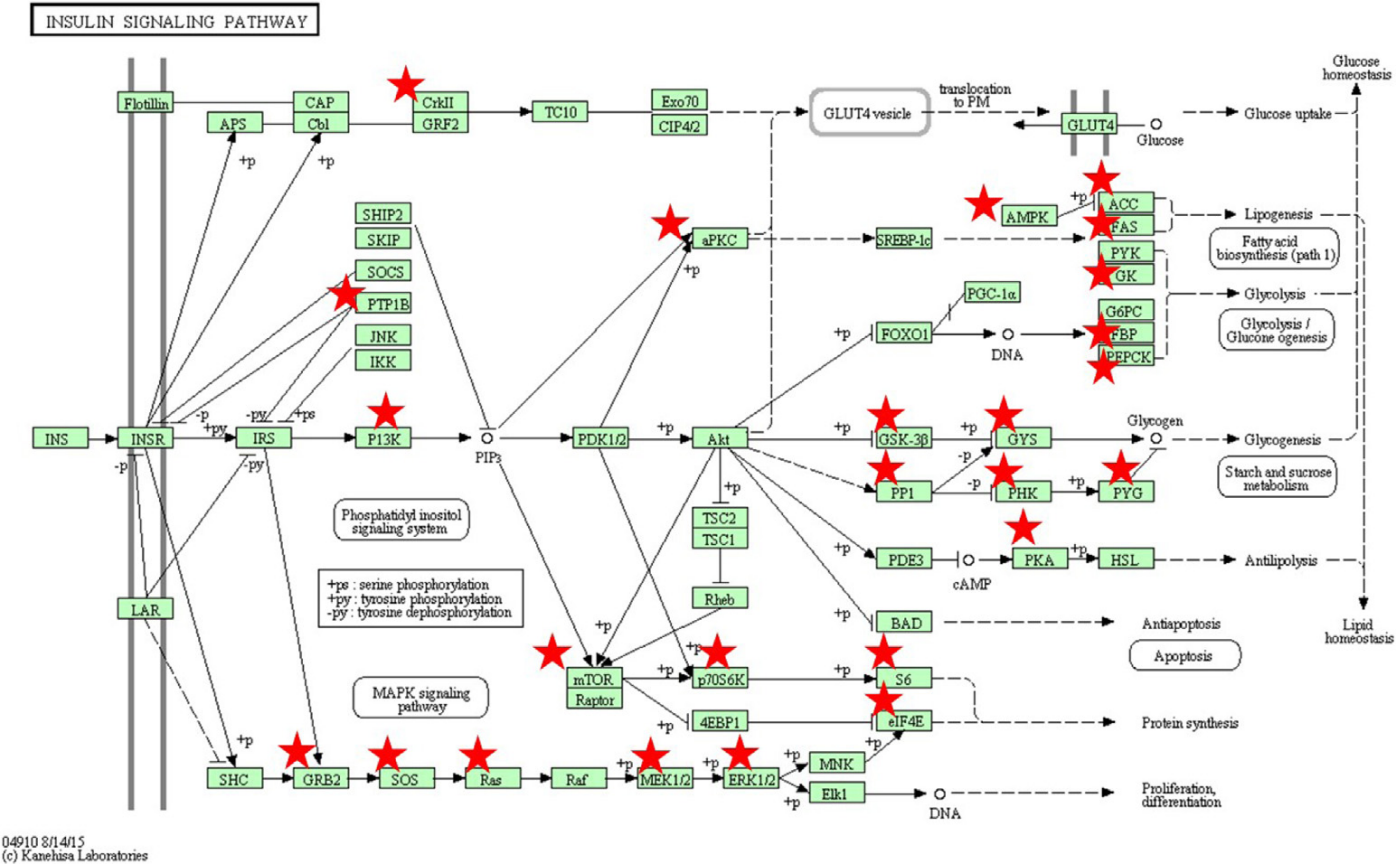
KEGG pathway for insulin signaling. Proteins identified in the MCF7 cell extract are marked on the diagram.

**Fig. 4:**
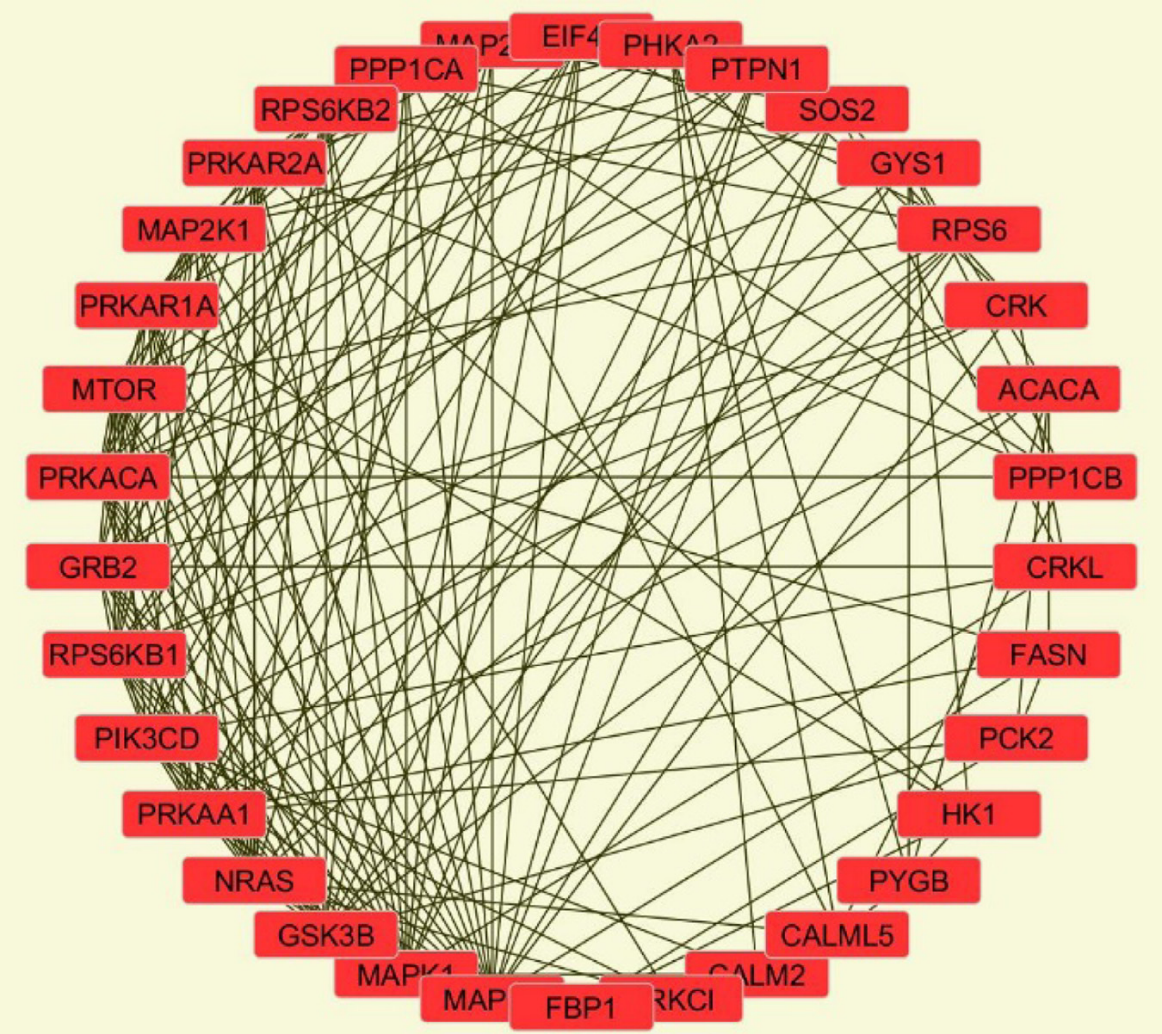
Cytoscape-generated protein-protein interaction network for identified insulin signaling proteins.

The MCF7 proteome data described in this manuscript include: (a) mass spectrometry RAW files deposited in PRIDE; (b) processed RAW files with the Thermo Electron Discoverer 1.4 software; (c) processed data with the DAVID (Database for Annotation, Visualization and Integrated Discovery [3], [4]) software package; and (d) processed data with the Cytoscape visualization tool set [5]. Fig. 1, Fig. 2, Fig. 3, Fig. 4 provide the sample preparation protocol, representative base-peak chromatograms for the 16 SCX peptide fractions, the KEGG (Kyoto Encyclopedia of Genes and Genomes [6]) insulin signaling pathway with identifiable proteins marked in red, and a Cytoscape-built protein–protein interaction network encompassing 34 insulin signaling proteins (see also link to interactive network). Supplementary Tables 1 and 2 provide Excel spreadsheets with lists of proteins (1898) and peptides (6802 unique, 12522 PSMs). Supplementary Tables 3 and 4 encompass the KEGG pathways and the GO (Gene Ontology) and functional annotation charts generated with DAVID. Supplementary Table 5 provides the list of interactions associated with the 34 insulin signaling proteins generated with STRING (Search Tool for the Retrieval of Interacting Genes/Proteins [7]).

## 2 Experimental design, materials and methods

### 2.1 Cell culture

MCF7 breast cancer cells were cultured in EMEM with FBS (10%) and insulin (10 µg/mL) at 37 °C in an incubator with 5% CO_2_. At 70–80% confluence the cells were detached by trypsinization, harvested and stored in a freezer at −80 °C.

### 2.2 Cell processing

The cells were lysed by rocking with RIPA buffer supplemented with protease and phosphatase inhibitors for 2 h at 4 °C. The final composition of the lysis solution was: 1 mL RIPA buffer, 100 µL protease inhibitor cocktail (104 mM AEBSF, 0.08 mM aprotinin, 2 mM leupeptin, 4 mM bestatin, 1.5 mM pepstatin A, 1.4 mM E-64), 100 µL NaF (100 mM), 50 µL Na_3_VO_4_ (200 mM) and 8.75 mL of ice cold water [1]. The cells were centrifuged at 15,000*g* (15 min, 4 °C), and the protein concentration in the supernatant was measured with the Bradford assay. The cell extract (1 mL containing 3 mg of proteins) was reduced with DTT (4.5 mM) in the presence of urea (8 M) for 1 h at 60 °C. The protein solution was then diluted 10 fold with NH_4_HCO_3_ (50 mM) and subjected to enzymatic digestion with trypsin at a protein:enzyme ratio of 50:1 w/w, overnight at 37 °C. The digestion reaction was quenched with 10 µL TFA/mL protein digest solution, and 300 µg of the protein digest was desalted with SPEC-PTC18 solid phase extraction tips. The sample was concentrated to a final concentration of 4 mg/mL with a vacuum centrifuge, and stored at −20 °C until further analysis by shotgun 2D-SCX/LC-ESI-MS/MS.

### 2.3 2D-SCX/nano-ESI-MS/MS

SCX prefractionation was accomplished with an 1100 HPLC system (Agilent) on a Zorbax Bio SCX Series II column (0.8 mm i.d.×5 cm column, Agilent) operated at 20 µL/min eluent flow. The eluent was H_2_O/CH_3_CN (95:5 v/v) supplemented with 0.1% HCOOH (solvent A), or 0.1% HCOOH and 500 mM NaCl (solvent B). The sample (16 µL injection containing 64 µg protein digest) was eluted from the SCX column in 16 fractions by a gradient consisting of: 100% A (0–5 min), 0–5% B (5–5.1 min), 5–20% B (5.1–35 min), 20–100% B (35–40 min), 100% B (40–50 min), 100 to 0% B (50–50.1 min) and 100% A (50.1–60 min). The first wash step (5 min) generated fraction 1. Fractions 2–15 were collected each for 3 min during the salt gradient. Fraction 16 was collected for 10 min at an eluent composition of mainly 100% B. Reversed phase nano-LC separations were performed with home-built capillary columns (100 µm i.d.×12 cm fused silica capillaries) packed with Zorbax SB-C18 (5 µm) particles (Agilent). The nano-LC column was fitted with a ~1 cm long nanospray emitter prepared from a fused silica capillary (20 µm i.d.×90 µm o.d.). The Agilent 1100 micro-HPLC system was modified with a home-built split/splitless setup to allow for the generation of solvent gradients in the nanoliter/min flow regime. Each SCX fraction was loaded separately on the nano-LC column (40 µL), and eluted by an eluent gradient at ~160–180 nL/min. Solvent A was prepared from H_2_O/CH_3_CN (95:5 v/v)+0.01% TFA, and solvent B from H_2_O/CH_3_CN (20:80 v/v)+0.01% TFA. The gradient consisted of: 0–10% B (0–1 min), 10–45% B (1–95 min), 45–60% B (95–110 min), 60–100% B (110–115 min), 100%B (115–120 min), 100 to 0% B (120–121 min) and 100% A (121–150 min).

### 2.4 Mass spectrometry

An LTQ linear ion trap mass spectrometer (Thermo Electron) was used for detection. Data acquisition occurred in data-dependent mode using 1 MS scan (5 microscans averaged) followed by 1 zoom scan (5 microscans averaged) and 1 MS^2^ on the top 5 most intense peaks. The zoom scan window was ±5 m/z. Dynamic exclusion parameters were set at repeat count 1, repeat duration 30 s, exclusion list size 200, exclusion duration 60 s and exclusion mass width ±1.5 m/z. Precursor ion fragmentation occurred by setting the collision induced dissociation (CID) parameters at isolation width of 3m/z, normalized collision energy 35%, activation Q 0.25 and activation time 30 ms.

### 2.5 Data processing

Raw data were analyzed with the Discoverer 1.4 software package (Thermo Electron) by using a *Homo sapiens* database with 20,199 entries downloaded from UniProt (January 2015). The database search parameters included: chemical and posttranslational modifications were not allowed, minimum and maximum peptide length was 6 and 144 amino acids, respectively, only fully tryptic fragments were considered for peptide matching, the number of allowed missed cleavage sites was 2, the precursor ion tolerance was 2 amu, the fragment ion tolerance was 1 amu, and the relaxed and strict false discovery rates (FDRs) were set at 3% and 1%, respectively. The quality of the data at the peptide level is verifiable from multiple tandem MS hits/peptide. The reliability of protein identifications can be inferred from the number of unique peptide hits/protein and FDRs set per user׳s preference and choice of search engine. The list of identified proteins was uploaded in DAVID to identify the KEGG signaling pathways and to generate the GO and functional annotation charts. All results were filtered with an EASE score of 0.1 [8]. The proteins matched to Kegg insulin signaling (34) were uploaded in STRING to extract the known protein–protein interactions related to this set of proteins. This list of interactions was uploaded to Cytoscape 3.4.0 to visualize the network of interactions in a degree sorted circle layout.

### 2.6 Reagents

Methanol and acetonitrile (HPLC grade) were purchased from Fisher Scientific, and deionized water (18 MΩ-cm) was generated in-house with a MilliQ ultrapure water system. MCF7 cells and cell culture reagents (EMEM, FBS, insulin, trypsin/EDTA) were purchased from ATCC, RIPA lysis buffer from Upstate, sequencing grade modified trypsin from Promega, protease inhibitors (NaF, Na_3_VO_4_) and other reagents (NaCl, TFA, HCOOH, TrisHCL, urea and DTT) from Sigma, and NH_4_HCO_3_ from Aldrich.

## Appendix A Supplementary material

**Supplementary Table 1**. List of proteins identified in MCF7 breast cancer cells stimulated with insulin (Thermo Discoverer 1.4 output).

**Supplementary Table 2**. List of peptides identified in MCF7 breast cancer cells stimulated with insulin (Thermo Discoverer 1.4 output).

**Supplementary Table 3**. KEGG signaling pathways identified in proliferating MCF7 cells stimulated with insulin (DAVID output).

**Supplementary Table 4**. GO and functional annotation charts for proteins identified in MCF7 cells stimulated with insulin (DAVID output).

**Supplementary Table 5**. List of interactions associated with the 34 insulin signaling proteins generated (STRING output).
