# Supplementary material for: Insulin stimulated MCF7 breast cancer cells: Proteome dataset
Source: Data Brief. 2016 Sep 22;9:579–84. doi: 10.1016/j.dib.2016.09.025 (PMC5064990; doi:10.1016/j.dib.2016.09.025)
Supplement: Supplementary file 1 — Supplementary material [file mmc7.pdf]

VirginiaTech
College of Science

Iuliana M. Lazar
Associate Professor
Department of Biological Sciences
1981 Kraft Drive, 2052 ILSB
Blacksburg, Virginia 24061
Phone: 540-231-5077; Fax: 540-231-9307
E-mail: lazar@vt.edu

September 4, 2016

Editorial Office
Data in Brief

We are re-submitting the revised version of our manuscript for publication in Data in Brief entitled "Insulin Stimulated MCF7 Breast Cancer Cells: Proteome Dataset." The authors are Hetal Sarvaiya and Iulia M. Lazar. The editorial and reviewers' comments were addressed, detailed responses being provided in the attached document.

The data provided in this article describe the proteome profile of insulin-stimulated MCF7 breast cancer cells, and include both RAW and processed data files.

The authors declare no financial or commercial conflicts of interest.

We thank you for your time and consideration.

Sincerely,

Dr. Iuliana M. Lazar
Associate Professor

Invent the Future
